# Impact of HPV vaccination: health gains in the Italian female population

**DOI:** 10.1186/s12963-017-0154-0

**Published:** 2017-09-29

**Authors:** Andrea Marcellusi

**Affiliations:** 1 National Research Council (CNR), Institute for Research on Population and Social Policies (IRPPS), Via Palestro 32, 00185 Rome, Italy; 2 Department Accounting, Finance and Informatics, Kingston Business School Kingston University, London, UK; 3 Economic Evaluation and HTA (EEHTA), CEIS, Faculty of Economics, University of Rome “Tor Vergata”, Rome, Italy

**Keywords:** HPV, Burden of disease, CIN1, CIN2–3, Anogenital warts, Cervical cancer, QALY, Daly

## Abstract

**Background:**

Human papillomavirus (HPV) is the leading cause of cervical cancer and other malignant and benign neoplastic lesions. HPV vaccination has three potential goals: to prevent transmission, infection, and disease. At present, there are no available data about health consequences of HPV immunization in Italy. The aim of this study is to evaluate the effect of current HPV vaccination strategy in Italy.

**Methods:**

A multistate morbidity-mortality model was developed to estimate the infection process in a theoretical cohort of Italian women. The Markov process considered nine health states (health, anogenital warts, grade 1 and grade 2/3 cervical intraepithelial neoplasia, cervical cancer, anal cancer, death due to cervical cancer, anal cancer and other causes), and 26 transition probabilities for each age group. The model was informed with the available data in national and international literature. Effectiveness of immunization was assumed considering a literature review pertaining to models and vaccination coverage rates observed in Italy. Life expectancy (e_x_), Quality-Adjusted Life Years (QALYs), Disability-Adjusted Life Years (DALYs), and attributable risk (AR) were estimated for no intervention (cervical cancer screening) and vaccination strategies scenarios.

**Results:**

The model showed that in a cohort of 100,000 Italian women the e_0_ is equal to 83.1 years. With current HPV vaccination strategy the e_0_ achieves 83.2 (+0.1) years. When HPV-related diseases are considered altogether, the QALYs increase from 82.7 to 82.9 (+0.2 QALYs) with no intervention and vaccination strategies respectively. DALYs decrease by 0.6 due to vaccination. Finally, AR is equal to 93 and 265 cases per 100,000 women in population and not vaccinated, respectively.

**Conclusion:**

When mortality due to cervical cancer is considered, HPV vaccination seems to have a low impact on health unit gains in the Italian female population. Conversely, when several HPV-related and cancer morbidity conditions are included, the effect of vaccination becomes quite remarkable.

**Electronic supplementary material:**

The online version of this article (10.1186/s12963-017-0154-0) contains supplementary material, which is available to authorized users.

## Key message

1. *The objective of this study is to estimate the impact of HPV-related diseases on the Italian population, in terms of health conditions and death risk. Furthermore, it evaluates the role of the Italian prevention system on health improvement, both in terms of mortality and morbidity.*
2. *A multistate morbidity-mortality model was developed to estimate the infection process in a theoretical cohort of Italian women. The Markov process took into consideration nine health states (health, anogenital warts, grade 1 and grade 2/3 cervical intraepithelial neoplasia, cervical cancer, anal cancer, death due to cervical cancer, anal cancer and other causes), in addition to 26 transition probabilities.*
3. *The results showed that in a cohort of 100,000 Italian women the e*_*0*_ *is equal to 84.31 years. With current HPV vaccination strategy the e*_*0*_ *achieves 84.36 (+0.05) years. When HPV-related diseases are considered altogether, the QALYs increase from 83.9 to 84.1 (+0.2 QALYs) with no intervention and vaccination strategies respectively. DALYs decrease by 0.6 due to vaccination. Finally, AR is equal to 0.08 and 0.29 in population and not vaccinated, respectively.*
4. *At present this work is the first model trying to evaluate the actual effect of vaccination on women’s health in Italy. The study considers the actual vaccination coverage nationwide, along with the distribution of different types of vaccines at regional level. It projects a fictitious cohort of women in order to evaluate the impact on morbidity and mortality trajectories of these individuals.*

## Background

Human papillomavirus (HPV) is probably the most common sexually transmitted viral infection worldwide [1]. It is well established that HPV is the causative factor in most cases of cervical cancer [2, 3]. High-risk oncogenic variants of HPV, specifically genotypes 16 and 18, account for approximately 75% of all cervical carcinomas [4]. However, HPV is involved both in the etiopathogenesis of invasive cervical cancer and in other malignant and benign neoplastic lesions that affect the vulva, vagina, anus, penis, head and neck, respiratory tract [recurrent respiratory papillomatosis (RRP)], and external anogenital area (genital warts acuminate) [2, 5–9].

HPV triggers about 600,000 cases of cervical cancer annually, cancer of the vulva, vagina, anus, penis, head and neck, as well as non-malignant neoplastic diseases, such as anogenital warts and recurrent respiratory papillomatosis (RRP), with an impressive medical and economic burden [10]. Italian data suggest the total direct cost associated with the annual incident cases of the nine HPV-related conditions (invasive cervical cancer, cervical dysplasia, cancer of the vulva, vagina, anus, penis, and head and neck, anogenital warts, and RRP) is estimated to be €528.6 million, with a plausible range of €480–686 million [11, 12].

Two vaccines (Cervarix and Gardasil) are currently available and utilized, providing protection against HPV genotypes 16 and 18. However, there are some differences between the vaccines. Gardasil, in fact, offers an additional protection against genotypes 6 and 11, which actually cause over 90% of anogenital warts and virtually all cases of RRP in both sexes [7, 8, 13].

Although the efficacy and cost-effectiveness of these prevention measures have been verified [14–16], no studies were conducted aimed at quantifying the impact of HPV-related diseases on women’s health in terms of mortality and morbidity in Italy. When the mortality profile is characterized by chronic diseases (second stage of epidemiological transition [17]), the analysis of mortality no longer suffices for a correct evaluation of population’s health. Consequently in a demographic stage, characterized by an increase in life expectancy and a significant growth in chronic pathologies, it is no longer possible to consider only the standard life table of a specific population, but it is necessary to analyze the morbidity profile of the population itself [18–21].

Different demographic tools allow the study of health and the impact of health care assistance. The life tables are one of the oldest tools used in demographic analyses and generally represent the starting points of demographic studies. The life tables describe the evolution of mortality in a single cohort of newborns, due to one or more death causes. However, a cohort may evolve in multiple death causes or health states (disease). In order to be investigated, these factors need an evolution of the traditional life tables. When we want to consider different death causes and disease and disability, like in the evaluation of HPV-prevention strategies, one of the most widespread methods to estimate life tables is the multistate Table [20–23].

The objective of this study is to estimate the impact of HPV-related diseases on the Italian population, in terms of health conditions and death risk. Furthermore, it evaluates the role of the Italian prevention system on health improvement, both in terms of mortality and morbidity.

## Methods

A multistate life table was developed in order to estimate trajectories of individuals who, in the course of time and age, move across health states through different transition probabilities. The multistate tables are based on the same assumptions of a Markov process, therefore:

- *The health states considered in the model are thorough and mutually exclusive.*
- *The probability to move from one health state to following states only depends on (is conditioned on) the health state of the individual at transition time (memoryless process).*
- *Transition probabilities are steady over time.*

The transition probability from state *i* to state *j* in the age interval *x* , *x* + *n* is initially represented by a central rate (*M*_*x*_), and then converted into transition probability *P*_(*x*, *n*)_, by using the method proposed by Rogers and Ledent in 1976 [24] and widely shared in the literature [25–27] (see Additional file 1).

### Model structure

**Fig. 1:**
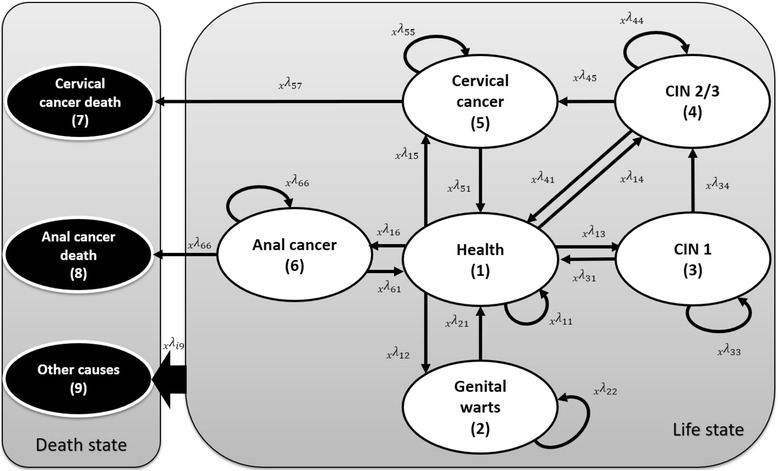
Markov process multistate tables: transitions at age x – adjustment of conceptual model

The disease evolution considered in the model represents a combination of the natural history of HPV-related diseases, with the health care assistance provided to those patients who may be diagnosed. This model, unlike what has been done in the literature, assumes that the transition probability from the health state to the other disease states are based on a more pragmatic version of the incidence rate (the real world history of the disease) and not on the virtual incidence rate (the natural history of the disease). Figure 1 shows a good approximation of this combination and includes the possibility for patients to move directly from the health state (state 1) to one of the considered morbidity states. For example, it is possible to move from anal and cervical cancer states (state 5 and 6 respectively) to the state of death for anal/cervical cancer (state 7 and state 8 in relation to the cause). Finally, depending on the age, specific mortality risks were applied using Italian life Tables [28].

#### Epidemiological data

Once the model structure is defined, it is necessary to identify the transition probabilities by age among the different states being considered. There are no secondary data that are referred to HPV-related diseases considered in this study. However, different studies have been conducted and provide scientific data concerning the burden of the disease on the Italian population and the progression and treatment of the considered diseases [11, 14]. Specifically, Baio et al. (2012) [11, 12] carried out a systematic review of the literature that identifies the prevalence and incidence of the main HPV-related diseases in Italy. Conversely, Favato et al. [14] developed a cost-effectiveness model, comparing two alternative prevention strategies (Screening vs. Screening + quadrivalent vaccine) in Italy. Starting from these studies, the probabilities described in Table 1 have been estimated considering age, disease state, and cause of death (see Additional file 1 for detail).

**Table 1:** Transition probability by age

Parameter	Description and parameters used to estimate transition probabilities	Source
Transitions from the health state (State 1)		
_*x*_ *λ*_11_	Probability of permanence in healthy state without onset of HPV-related diseases (addition to 1 of the remaining probabilities from state 1) 1 − _*x*_ *λ*_12_ − _*x*_ *λ*_13_ − _*x*_ *λ*_14_ − _*x*_ *λ*_15_ − _*x*_ *λ*_16_ − _*x*_ *λ*_18_	Complementary probability
_*x*_ *λ*_12_	Probability of genital warts onset = incidence of diagnosis by age in genital warts	[11, 14, 59, 63, 64]
_*x*_ *λ*_13_	Probability to contract a CIN 1 = incidence of diagnosis by age of CIN 1	[11, 65–67]
_*x*_ *λ*_14_	Probability to contract a CIN 2/3 = incidence of diagnosis by age of CIN 2/3	[11, 65–67]
_*x*_ *λ*_15_	Probability of cervical cancer onset by age = incidence of cervical cancer by age (assuming all cancers are diagnosed)	[60]
_*x*_ *λ*_16_	Probability of anal cancer onset by age = incidence of anal cancer by age (assuming all cancers are diagnosed)	[60, 68]
Transitions from the genital warts state (State 2)		
_*x*_ *λ*_22_	Probability of permanence of genital warts by age = Recurrence rate of genital warts by age between age *x* − 1 *and x*	[11, 59, 69]
_*x*_ *λ*_21_	Probability of healing from genital warts = 1 − _*x*_ *λ*_22_ − _*x*_ *λ*_28_	Complementary probability
Transitions from state of CIN 1 (State 3)		
_*x*_ *λ*_33_	Probability of permanence of CIN 1 by age =1 − _*x*_ *λ*_31_ − _*x*_ *λ*_38_	Complementary probability
_*x*_ *λ*_34_	Transition probability from CIN 1 to CIN 2/3 = progression rate of the disease by age from state CIN 1 to CIN 2/3	[11, 70]
_*x*_ *λ*_31_	Probability of healing from CIN 1 by age = spontaneous regression rate of the disease by age. In this case women are only observed and not treated.	[11, 70–72]
Transitions from state of CIN 2/3 (State 4)		
_*x*_ *λ*_44_	Probability of permanence in CIN 2/3 =1 − _*x*_ *λ*_41_ − _*x*_ *λ*_48_	Complementary probability
_*x*_ *λ*_41_	Probability of healing from CIN 2/3 by age = efficacy rate of health care intervention on diagnosed women (Constant by age)	[11, 70–72]
_*x*_ *λ*_45_	Transition probability from CIN 2/3 to CCU state by age for lack of efficacy of health care intervention = progression rate of the disease by age	[11, 66, 70–72]
Transitions from Cervical cancer state (State 5)		
_*x*_ *λ*_55_	Probability of permanence of CCU state by age = recurrence rate (recurrence of disease) of CCU	[73, 74]
_*x*_ *λ*_51_	Probability of healing from cervical cancer state by age = 1 − _*x*_ *λ*_55_ − _*x*_ *λ*_57_−_*x*_ *λ*_59_	Complementary probability
_*x*_ *λ*_57_	Probability of death from cervical cancer by age for ill subjects =1 − _*x*_ *λ*_59_ − 5 − *year survival by age*	[28, 75]
Transitions from Anal Cancer (State 6)		
_*x*_ *λ*_66_	Probability of permanence in CCU state by age = recurrence rate of anal cancer (recurrence of disease)	[68]
_*x*_ *λ*_61_	Probability of healing from cervical cancer state by age =1 − _*x*_ *λ*_66_ − _*x*_ *λ*_67_−_*x*_ *λ*_69_	[28, 75]
_*x*_ *λ*_68_	Probability of death from anal cancer by age for ill subjects =1 − _*x*_ *λ*_69_ − 5 − *year survival by age*	Complementary probability
Transitions to death for other causes (State i - > State 9)		
_*x*_ *λ*_*i*9_ *i* = 1, 2, 3, 4, 5,6	Probability of death by age from any state for other causes except for cervical cancer = Probability of general death by age	[28]

Legend: *i* = state of origin; *j* = state of destination; x = Age group: 0–4, 5–9, 10–14, 15–19, 20–24, 25–29, 30–34, 35–39, 40–44, 45–49, 50–54, 55–59, 60–64, 65–69, 70–74, 75–79, 80–84, ≥85;

### Burden of disease index (DALYs, QALYs, and impact measures)

Different indicators may estimate the impact of a chronic disease on women’s health. The most commonly widespread indicators used in economic evaluations [29], World Health Organization (WHO) [30, 31] and the National Institute of Health (ISS) [32] have been utilised in this study.

The quality-adjusted life years (QALY) are a measure of health widely used in the economic evaluations of health care interventions [29]. The rationale for this indicator relies on the term “utility,” indicating the well-being of an individual using a health care service [33]. According to this assumption, a weight related to the morbidity lived by the individual is assigned to each health state. The utility scale of these states is given by values worth 0 (minimum preference corresponding to death) and 1 (maximum preference corresponding to perfect health).

**Fig. 2:**
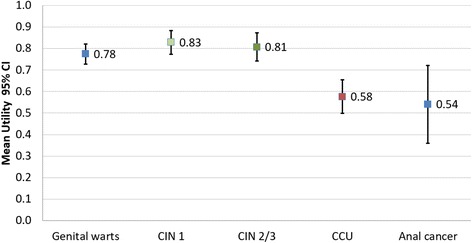
Estimate of utilities by pathological state (mean values and 95% CI) [35]

However, recently Marcellusi et al. [34] estimated the utilities expressed by women and men who lived or are living HPV-related pathologies, through the Time Trade Off questionnaire (TTO). The authors interviewed about 60 patients (an ideal number due to the variability of the detection tool recorded in the validation study [33]), divided by pathological group. Specifically, the inquired pathological conditions were the following: genital warts, cervical intraepithelial neoplasia (CIN) grade 1 and 2/3, cervical and colorectal-anal cancer, head and neck squamous cell carcinoma (HNSCC), and ano-genital warts (AWS). Figure 2 reports the results of the estimated utilities only for the diseases considered in the multi-state model.

The concept of DALY was introduced for the first time in 1993 by the World Bank in the World Development Report [35]. In general, DALYs can be represented as the sum of years of life lost due to the mortality caused by a disease (YLL) and the years lost due to disability (YLD). YLLs correspond to the number of specific deaths due to cause *i*, multiplied by the life expectancy at age of death. As far as the years lived with disability are concerned, the method is similar to that used to estimate QALYs. The disability is measured as the product of the incident cases with the disease, quality of life of the pathological condition, and duration of the disease.

In order to estimate the impact of HPV prevention on population health, specific attribution measures were estimated [32]. The assumption of these measures is simple: if behavior (exposure) is associated with a disease, how would the disease frequency change if the exposure disappeared? Or, more realistically, if the exposure frequency changed [32]?

In our model the prevention factor is the anti-HPV vaccine for 12-year-old girls. The attributable risk in the exposed (*RA*_*E*_) may be estimated as the difference between the incidence rates in the exposed and unexposed. Therefore, *RA*_*E*_ represents the fraction of HPV-related diseases that may be prevented with the vaccine. A further impact indicator is represented by the attributable risk in the population (*RA*_*P*_), obtained by the difference between the incidence in unvaccinated people and the incidence in the population. In this case, *RA*_*P*_ is the number of avoided cases thanks to the primary prevention (vaccination) implemented in Italy.

### Estimate of effectiveness in vaccination strategies

The parameters available in Italy to be considered for the implementation of the effectiveness data of the two vaccines are the following:

1. *Vaccination coverage: number of actually vaccinated subjects with the three doses, on the total of 12-year-old girls for whom free anti-HPV vaccine is available in Italy;*
2. *Number of vaccinated subjects with Bivalent vaccine (coverage for HPV 16 and 18) and Quadrivalent vaccine (coverage for HPV 6, 11, 16 and 18);*
3. *Effectiveness of vaccination on HPV preventable serotypes;*
4. *Number of avoided cases thanks to the reduction of subjects contracting the virus.*

With reference to points 1 and 2, a cohort of women (born in 1998) who benefited from free vaccination in 2008 (when aged 11) has been considered. Therefore, the results of the model will refer to a fictitious cohort of women that have experienced the vaccination coverage rate and distribution of the two types of vaccination identical to this cohort. Table 2 summarizes coverage data and the type of administered vaccination reported by the ISS [36].

**Table 2:** Distribution of population actually vaccinated by type of vaccine and region – Italy 2009 (cohort 1998) [36]

Region	Vaccination [36]	Vaccine coverage [36] %	Resident [76] 12-year-old girls absolute values	Quadrivalent vaccinated absolute values	Bivalent vaccinated absolute values
Valle d’Aosta	Bivalent	73.8	483	0	356
Piemonte	Quadrivalent	64.6	17,048	11,013	0
Liguria	Bivalent	72.8	5625	0	4095
Lombardia	Bivalent	64.7	39,751	0	25,719
PA Trento	Bivalent	63.4	2534	0	1606
PA Bolzano	Quadrivalent	25.5	2534	646	0
Veneto	Quadrivalent	74.9	20,297	15,202	0
Friuli VG	Quadrivalent	71.7	4546	3259	0
Emilia Romagna	Quadrivalent	75.6	15,898	12,019	0
Toscana	Bivalent	84.3	13,705	0	11,553
Marche	Bivalent	72.7	6379	0	4638
Umbria	Bivalent	80.3	3480	0	2794
Lazio	Quadrivalent	64.8	23,324	15,114	0
Abruzzo	Quadrivalent	73.8	5681	4193	0
Molise	Quadrivalent	65.9	1503	990	0
Campania	Quadrivalent	62.6	33,223	20,798	0
Basilicata	Quadrivalent	82.4	2814	2319	0
Puglia	Quadrivalent	81.4	20,848	16,970	0
Calabria	Bivalent	69.2	10,294	0	7123
Sicilia	Quadrivalent	55.3	26,522	14,667	0
Sardegna	Bivalent	84.7	6825	0	5781
TOTAL			263,313	117,190	63,666

With reference to point 3, the evaluation of vaccine effectiveness and tolerability has been the object of an extended and deep clinical research program, involving about 21,000 women aged between 16 and 26 years, and over 2500 adolescents aged between 9 and 15 years. In double-blind, placebo-controlled multicentric studies of phase II and III, the vaccine administered in three doses (according to a scheduling of 0, 2, and 6 months) reduced by 100% (95% confidence intervals [CI]: 91.0–100.0%, *p* < 0.001) the development risk of pre-cancerous high-grade lesions (CIN 2/3) of adenocarcinoma in situ and invasive cancer, associated with the viral types included in the vaccine, over a follow-up period of 2–2.5 years [37–43]. With reference to external genital lesions (genital warts, vulvar, vaginal grade 1/3 lesions), none of the vaccinated women (*n* = 2261) developed such lesions, compared to the 40 recorded cases out of 2279 women belonging to the placebo group (*p* < 0.001) [42].

In order to summarize the effects of vaccination by age, reference was made to the study of Favato et al. [14] in which, as previously mentioned, vaccine effectiveness was projected on HPV-related events. In particular, based on main clinical randomized trials of the quadrivalent vaccine [42, 44–48] and the support provided by a board of clinical experts, the authors projected the effectiveness of this vaccine on a standard population, using a Bayesian approach. Furthermore, the authors assumed that the vaccine reduced the transmission of over 10 different types of non-vaccinable HPV (31, 33, 35, 39, 45, 51, 52, 56, 58, 59), causing about 20% of cervical cancers [49]. As a consequence, the vaccine reduces by 32.5% (95% CI: 6.0–51.9%) the development of pre-cancerous stages (CIN 2/3), associated with 10 kinds of non-vaccinable viruses [47, 48]. This effect is defined *cross-protection* and has been widely discussed and demonstrated in the literature [47, 48, 50, 51].

**Fig. 3:**
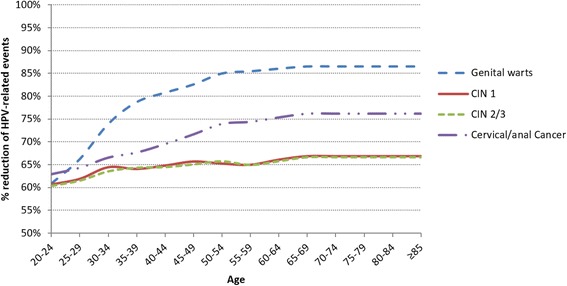
Effectiveness of vaccination by age and pathological condition – reduction rate of events by age $\left({}_x^{\ast }{\theta}_{\mathrm{ij}}\right)$ [14]

Starting from the published model, the reduction effects of HPV-related events have been extrapolated, considering a vaccinated cohort and an unvaccinated one. As reported in Fig. 3, the effectiveness of quadrivalent vaccination is distinguished by age. In fact, from the introduction of the vaccine - 14 years for the first cohort - we can expect that its effectiveness increases due to the effects on recurrences and reaches a plateau after the disease peaks.

Finally, reduction rates of HPV-related events $\left({}_x^{\ast }{\theta}_{\mathrm{ij}}\right)$ have been applied to transition probabilities from the health state (state 1) to the genital warts disease states, CIN 1, CIN 2/3 and CCU:

$$ {{}_x\lambda}_{1j}^{Vacc}={{}_x\lambda}_{1\mathrm{j}}\times {{}_{12}\pi}_{1\mathrm{j}}\times {{}_x\theta}_{1\mathrm{j}}\kern1em for\kern1.25em j=2,3,4,5 $$

where ${{}_x\lambda}_{1j}^{Vacc}$ represents the transition probability by age *x* in the model in which HPV vaccination is considered, _12_ *π*_1j_ is the percentage of patients actually vaccinated and _*x*_ *θ*_1j_ is the reduction rate of HPV-related events estimated by the BEST study [14].

With reference to the two types of vaccination, it has been assumed that the efficacy towards genital warts only occurs in the proportion of women who submitted to quadrivalent vaccination. Regarding the anal cancer, the model assumes that the vaccination effectiveness was the same of the cervical cancer considering that the HPV genotypes associated with the two cancers are similar (HPV 16 and 18) [11, 52].

### Sensitivity analysis

In order to verify the uncertainty of the model, a one-way deterministic sensitivity analysis (DSA) was conducted, changing each parameter with a plausible interval on the main important parameters of the model. This kind of analysis allowed identification of the parameters that mostly influenced the change of the final estimation of QALYs gained or DALYs lost due to vaccination strategy. The definition of possible may vary from model to model, but normally it is reasonable to change the parameters according to a plausible interval or the data available in the literature.

More specifically, the model parameter tested in this DSA were:

- *Screening variation: the model assumes that cancer screening and management are constant over time in the base-case. In order to test the variability on this parameter, a deterministic sensitivity analysis was performed by simulating a specific scenario in which the incidence rates of CIN1/2/3 were higher/lower than the base case (due to an increment/decrement effect of screening program ± 20%) and, consequently, this reduce/increase the incidence rate of cervical cancer;*
- *Vaccine efficacy: ±20% of*_*x*_ *θ*_1*j*_ *parameter;*
- *Coverage rate: ±20% of the coverage rate*_*x*_ *π*_1*j*_ *;*
- *Vaccine type: the minimum scenario considers the vaccination with the only HPV2 vaccine and the maximum scenario with only HPV4 vaccine;*
- *Utilities: the minimum scenario considers the utilities estimated by Baio* et al. [52] *(lower credibility interval) and the maximum scenario considers the utilities considered by Elbasha* et al. [53]*.*

## Results

### Impact of HPV-related diseases on unvaccinated women

The mortality-morbidity table in case of lack of vaccination was estimated and the main indicators are represented in Table 3. Specifically, the model outlined that 28 women out of 1000 (28.037 × 100,000 women) live a HPV-related pathological condition (genital warts, CIN 1, CIN 2/3, CCU and death from CCU). With reference to life expectancy at birth, a woman following the behavior of the fictitious cohort considered in the model, may expect to live 83.1 years, of which 1.65 years will be in one of the HPV-related disease conditions.

**Table 3:** Main indicators for type of vaccination strategies - fictitious cohort of Italian women with vaccination coverage and vaccine distribution of 1998 cohort vaccinated in 2009 in Italy (Radix of the Tables 100,000 women) – Year 2012

Health state	Subjects per state	Lived years per health state	Lived QALY per woman	Life expectancy at birth by health state
No vaccination				
Healthy	71,963	8,146,560	81.466	81.466
Genital warts	19,894	121,849	0.950	1.218
CIN 1	3679	19,458	0.162	0.195
CIN 2/3	3004	18,195	0.147	0.182
Cervical cancer	825	4123	0.024	0.041
CC death	296	–	–	–
Anal cancer	195	976	0.006	0.010
AC death	145	–	–	–
Total	100,000	8,311,161	82.754	83.112
Vaccination				
Healthy	84,421	8,222,681	82.227	82.227
Genital warts	11,180	69,003	0.538	0.690
CIN 1	2056	10,873	0.090	0.109
CIN 2/3	1682	10,187	0.083	0.102
Cervical cancer	365	1826	0.011	0.018
CC death	131	–	–	–
Anal cancer	94	471	0.003	0.005
AC death	72	–	–	–
	–	–	–	–
Total	100,000	8,315,042	82.951	83.150

**Fig. 4:**
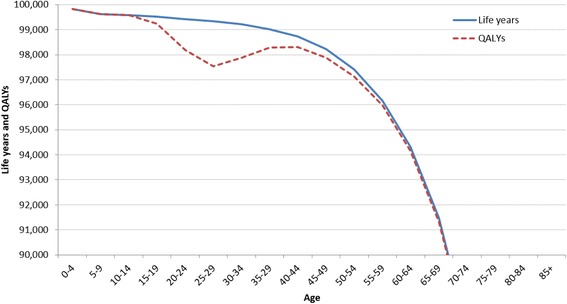
QALYs lived by the cohort vs. Lived Years (L_x_) by age – fictitious cohort of Italian women (table root 100,000 women) – Year 2012

Comparing the QALYs lived by the fictitious cohort of unvaccinated women (82.75 QALYs) with the life expectancy of the same women at birth (83.1 years), the model concludes that HPV-related diseases cause a burden on women’s health of 0.35 years of perfect health lost due to HPV-related diseases. This aspect is represented by the area between the two curves of Fig. 4 in which the curves represent, respectively, the years lived by the cohort of the table (continuous line) and the QALYs lived by the same population (red dotted line). As reported in Fig. 4, the higher decrease of life quality compared to years lived is observed between 20 and 45 years of age. In fact, at these ages, the prevalence of HPV-related diseases considered in the model is concentrated. Specifically, genital warts are the most prevailing conditions (19.894 cases out of 100,000 women) with a higher incidence in younger ages. Pre-cancerous conditions (CIN) later arise, impacting on the quality of life of women aged between 40 and 50.

**Fig. 5:**
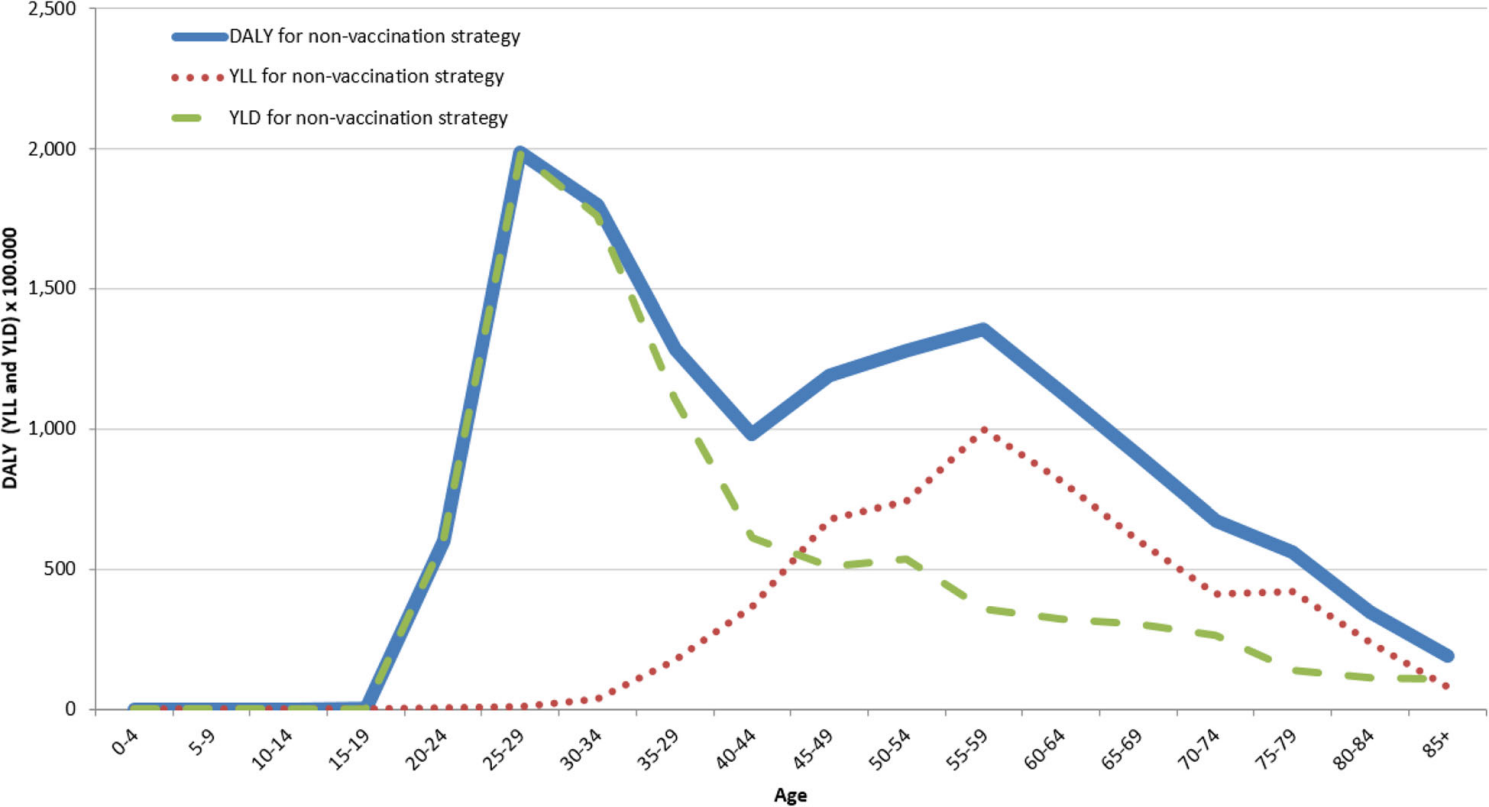
DALYs lived by the fictitious cohort of Italian women (radix of the Tables 100,000 women) – Year 2012

The estimated curves of the life years lived with disabilities (YLDs), the years of life lost (YLLs), and the disability-adjusted life years (DALYs) for the cohort due to HPV-related diseases considered in the increment-decrement model are represented in Fig. 5. Specifically, our model estimated a sharp rise of both DALYs (continuous curve) and years lived with disability (YLDs) after 15 years of age. However, even if DALYs decline after 45 years of age they remain somewhat high due to the impact of the years of life lost. In fact, the higher prevalence of pathological conditions in the first stage of women’s life, affects the worsening of quality of life (genital warts and CIN) and increases the number of DALYs. However, in the age groups over 40, the years of lost life due to the disease (cervical cancer and anal cancer) keep the number of DALYs lived by the cohort women high.

### Effects of anti-HPV vaccination on women’s health

The burden of HPV-related diseases considering the women included in the vaccination strategy is reported in Table 3. On average the model estimates that 15 cases out of 1000 (15,579 out of 100,000) live a pathological condition. The years lived with the disease represent about 1.1% of the total (98,361 out of 8,315,042 years lived by 100,000 women) and the life expectancy is 83.15 years, of which 0.92 years lived with the disease. QALYs for vaccinated women are 82.95 (life years lived in perfect health) and the estimated morbid weight of HPV-related diseases corresponds to 0.2 QALYs (84.95 QALYs vs. 83.15 years of life expectancy at birth).

Consequently, by comparing the cohort of unvaccinated women with the cohort of women who may benefit from the vaccination (Table 4), we obtain 3881 additional years for 100,000 women. Also, the lifespan lived in a healthy life would increase by approximately 76,121 years for 100,000 women and by more than 19,000 QALYs. The decrease of disability-adjusted life years is of about 6900 DALYs (40% of the decrease due to years of life lost).

**Table 4:** Comparison of main comparison indicators between vaccinated cohort and unvaccinated one – fictitious cohort of Italian women with vaccination coverage and vaccine distribution of 1998 cohort vaccinated in 2009 in Italy (Radix of the Tables 100,000 women) – Year 2012

Simulations	Lived years	Healthy life years	QALY	DALY	YLL	YLD
Cohort of unvaccinated women (100,000 women)	8,311,161	8,146,560	8,275,447	14,686	5567	9119
Cohort of vaccinated women (100,000 women)	8,315,042	8,222,681	8,295,112	7712	2760	4952
Increases (Vaccinated – Unvaccinated)	3881	76,121	19,665	-6974	-2807	−4167

**Fig. 6:**
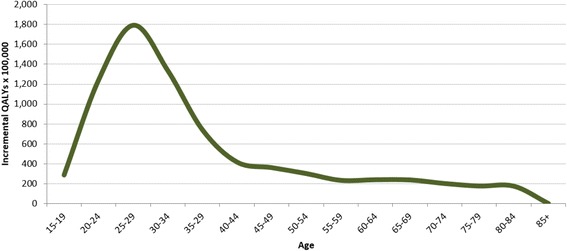
QALYs by the fictitious cohort of Italian women thanks to vaccination (radix of the Tables 100,000 women) – Year 2012

Furthermore, as represented in Fig. 6, QALYs gained by vaccinated women compared to unvaccinated ones are much higher for women aged 25 to 29 years with a peak at about 1800 incremental QALYs per 100,000. However, among women aged more than 40 years, the model estimates a lower value (about 200 QALYs per 100,000) that also remains constant in the remaining period. This effect by age is once again explained by the higher prevalence of genital warts (in numerical terms) that are more common among young people.

**Fig. 7:**
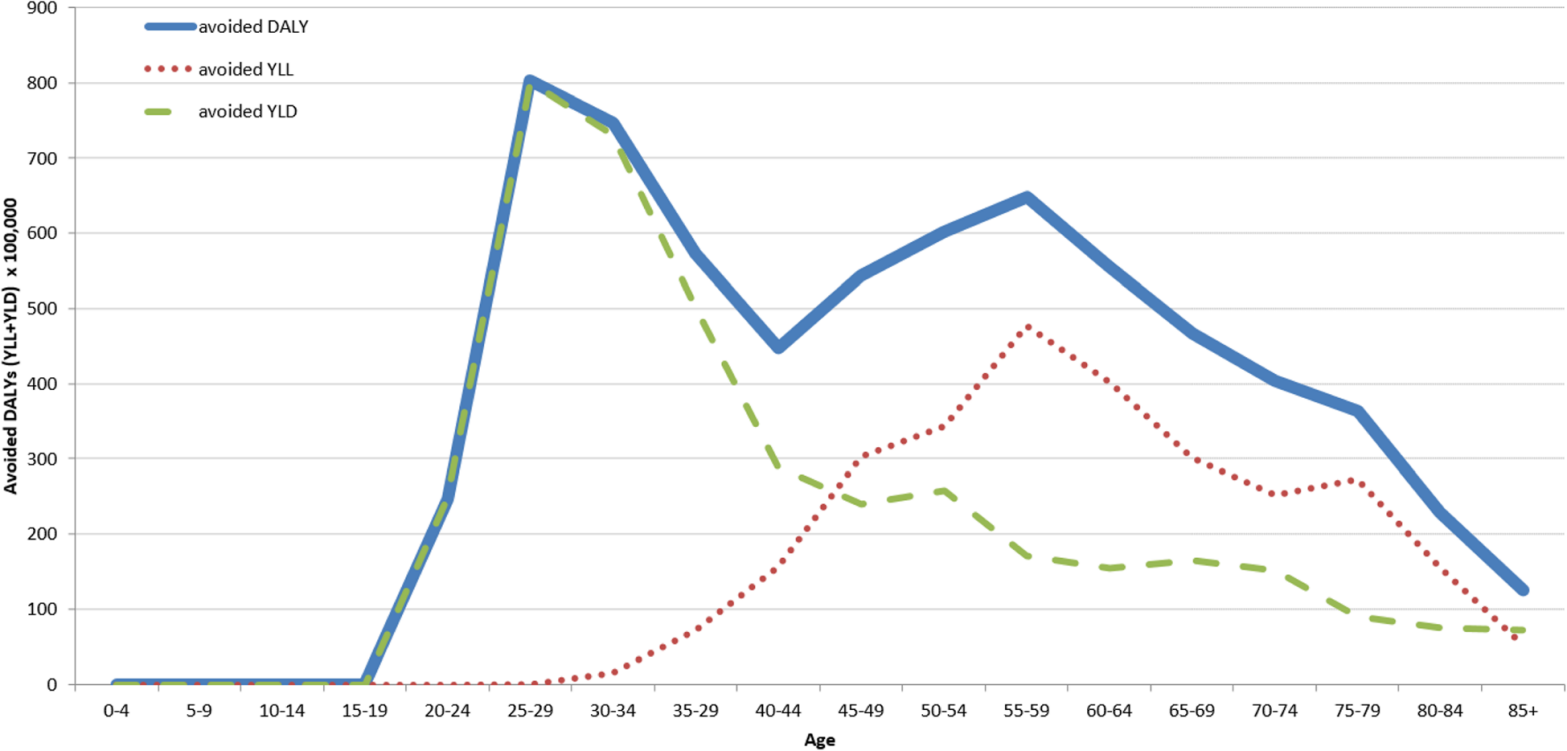
Avoided DALY by the fictitious cohort of Italian women thanks to vaccination (radix of the Tables 100,000 women) – Year 2012

The effect of age and genital warts can be also observed in the estimate of avoided DALYs as reported in Fig. 7. In fact, due to vaccination (represented by the green dotted line) and the consequently avoided YLD, a higher rate of disability is prevented in younger ages (<15 years). In older ages, vaccination contributes to the number of avoided YLLs and to the health gains in terms of avoided DALYs (Fig. 7).

**Fig. 8:**
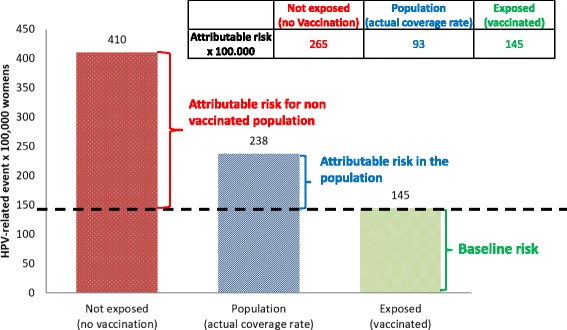
Impact measures of a fictitious cohort of Italian women (risk for 100,000 women) – Year 2012

Figure 8 summarizes the main impact of the measures analyzed in the multistate model. In particular, considering the anti-HPV vaccination as exposure factor, the model reports a risk of 145 events out of 100,000 women. In other words, assuming 100% vaccination coverage with maximum efficacy, there will be at least 1.5 cases out of 1000 women that we will not be able to avoid with prevention. These HPV-related events are due to the lack of vaccine effectiveness on total HPV strains that, even if combined, would be highly effective only in relation to HPV types 6, 11, 16, and 18, and consequently only to part of the considered diseases [11]. However, by comparing this value with the actual coverage rate, the model estimates 93 women out of 100,000 that represent the number of cases that could be avoided if vaccination coverage reached the entire population. In conclusion, in case of no vaccination, we estimate 265 cases out of 100,000 that could be avoided taking some preventive measures.

### Sensitivity analysis

**Fig. 9:**
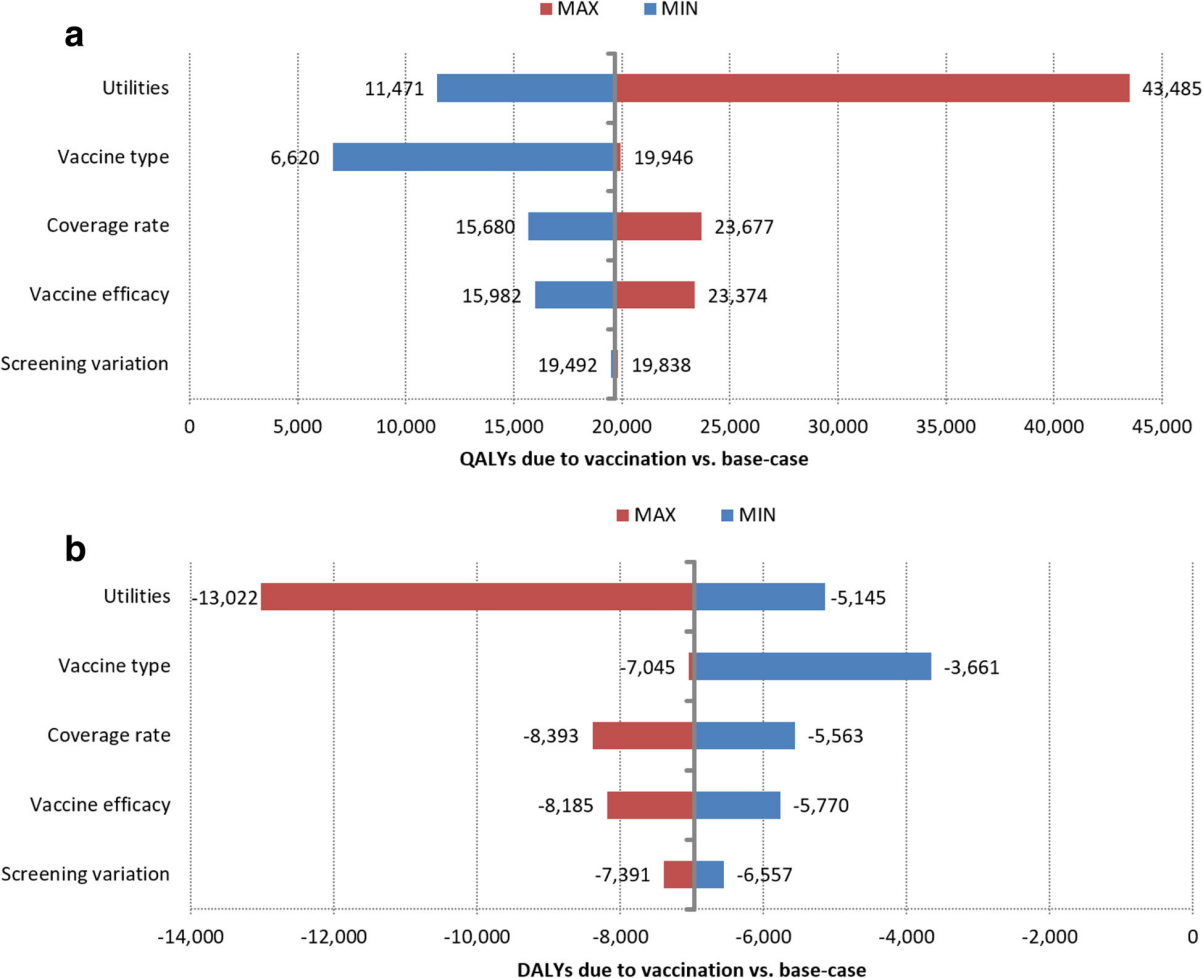
Deterministic Sensitivity Analysis results – tornado diagram QALYs (**a**) and DALYs (**b**)

Figure 9 reports the deterministic sensitivity analysis (DSA). The figure shows the variation of QALYs gained and DALYs lost due to the vaccination strategy (difference between vaccination scenario results and base case results) if such parameters were changed in the model and represents these variation (bar of the figure) compared to the same outcome in the principal analysis (y-axis of the graph).

The simulations shows that the most sensible parameters are the utilities associated with the disease states that have important impacts on both QALYs gained or DALYs lost due to vaccination strategy (Fig. 9). A variation in the utilities, as reported in the maximum scenario, decreases the number of QALYs gained from the base-case of 19,665 to −11,471. Conversely, considering the minimum scenario with lower utilities for each disease state, QALYs gained increase by 11.471 QALYs (42% respect to the base-base) (Fig. 9a). The same variation would affect the number of DALYs lost with a variation of approximately 13.000 DALYs for the maximum scenario and 5.000 DALYs for the minimum (Fig. 9b).

The DSA also shows that the vaccination type plays an important role on the HPV-related disease impact for women health. In fact, using the bivalent vaccine only, the model estimates an increase of “only” 6.620 QALYs compared to the base-case while the adoption of quadrivalent vaccine only could increase the number of QALYs gained vs. the base case of 19.946 years lived in perfect health.

A variation on the coverage rate, screening, and vaccine efficacy (± 20% respect to the base case analysis) showed a lower impact on the number of DALY lost: ± 7%, 6%, and 2% respectively if compared to the base-case results. The effect predicted to the model for deterministic sensitivity analysis corresponds to a variation of QALYs gained between ±19% for vaccine efficacy and ±20% for coverage rate while no impact was estimated for screening variation scenario (± 1% respect to the base-case analysis).

## Discussion

The aim of this model was to evaluate the actual effect of vaccination on women’s health in Italy. The analysis projects a fictitious cohort of women in order to evaluate the impact on morbidity and mortality trajectories of these individuals. Doing so, the study considers the actual vaccination coverage nationwide, along with the distribution of different types of vaccines at a regional level. As we know, the main objective of HPV prevention strategies is usually to reduce the number of cervical cancers (which are a direct consequence of the negative evolution of HPV). However, the impact of HPV on women’s health concerns both the negative evolution of cervical cancer (to death) and a number of diseases related to the virus. In addition to mortality, the model also included the analysis of the morbidity of HPV-related diseases in order to provide a more appropriate evaluation of the epidemiological burden of these pathologies and the possible impact of different vaccination strategies.

As evidenced by the study, primary prevention plays an important role in improving women’s health. Anti-HPV vaccination, in particular, may improve life expectancy, increase the quality of life, and reduce the disability of women that have experienced HPV-related diseases. Comparing the QALYs lived by the fictitious cohort of unvaccinated women (82.7 QALYs) with the life expectancy of the same women at birth (83.1 years), it is possible to notice that HPV-related diseases cause a burden on women’s health of 0.35 years of perfect health lost due to HPV-related diseases.

Different studies were conducted in order to evaluate the impact of different vaccination strategies in Italy. Merler and Ajelli (2013) [54] studied the anti-measles vaccination strategy on the Italian population during the last century. Similar to our work, the authors developed an infection transmission model considering a stationary population with a Markov structure that projects two scenarios: a) the natural history of the disease (base-case) and b) a simulation of the effect of vaccination and coverage rate registered in Italy for the same population. The Merler and Ajelli (2013) [54] study is similar to our model for the applied methodology and the perspective but differs in terms of objective. In fact, their study was aimed at evaluating the impact of measles on the decrease of fertility in Italy from a demographic perspective while our objective was to evaluate morbidity and mortality from a demographic perspective.

Guzzetta et al. (2014) [55] tried to estimate the progression of HPV infection and the clinical consequences through a mathematical model that considers different levels of sexual activity under the hypothesis of demographic equilibrium over time. Also in this case, the authors simulated different cohorts of female population considering different vaccination strategies. Similarly to our model, Guzzetta et al. compared a scenario with no vaccination vs. a 70% coverage rate of 12-year-old vaccinated female. In agreement with our results, Guzzetta et al. estimated a reduction of around 50% of cervical cancer cases after 80 years of simulation thanks to HPV vaccination. However, in addition to some methodological aspects that differentiate our work from that of Guzzetta et al. (multistate static approach vs. dynamic evolution of different cohort), our study has three main innovative aspects: a) a greater number of related diseases considered in the model (cervical cancer vs. cervical cancer, CIN 1/2/3, anal cancer, and genital warts) that have a considerable impact in terms of epidemiology and quality of life, [1, 11, 56] b) a combination of HPV 4 and HPV 2 vaccine as registered in the different Italian regions with different coverage rates (Guzzetta et al. consider the only HPV 2 vaccination with a constant coverage rate equal to 70%), and c) the chance to consider not only the disease event avoided but also the disease impact in terms of quality of life, disability, and avoided risk thanks to the primary prevention adopted in Italy in the previous years.

In the previous literature, the economic perspective has been frequently adopted for the evaluation of the impact of the HPV-related diseases. For example, the study of Favato et al. [14], simulates a different cohort of female population in Italy. However, also in this case, the model refers to HPV 4 vaccine only and considers results in terms of cost avoided and QALYs without any estimation in terms of expectancy, disability, or impact on life expectancy in good health. Indeed, the main objective of all models cited before was to project the virus effects on the female population. In our model, the estimation is based on diagnosis rate registered in the Italian population and estimation of the morbidity-mortality life table, considering the risk variation generated by the prevention strategy. The second approach represents a simplification of the real infectious disease progression but considers the most reliable data available relating to the diagnosis rate and not only the hypothetical natural history of the virus [57].

Some consideration about the economic consequences of our model is also warranted. Considering constant risk over time, we can estimate that in Italy the HPV vaccination strategy could reduce yearly cervical cancer cases from 3000 [11] to 1400 (considering the reduction estimated from our model). This reduction might have a positive impact in terms of quality of life and life expectancy and in terms of economic resources. In fact, we can estimate a yearly cost reduction of over €38 million (assuming a mean cost per treated case equal to €24.286 [11, 58]). Considering also the pre-cancerous disease state, we can estimate additional €10 million generated by the reduction of the 28,000 [11] yearly cases in Italy to around 16,400 cases of CIN 1/2/3. Finally, of the 62,000 genital warts estimated in Italy each year [11] the vaccination strategy could avoid over 37,000 cases per year generating a cost reduction of additional €18 million [11].

Obviously, this study has different limitations that have to be taken into account. First of all, currently available data are not always referred to national incidence data. They often refer to registry data distributed nationwide (AIRTUM Cancer Registry) or to previously published epidemiological data (BEST study to estimate effectiveness) [11, 12, 14, 59, 60]. However, as of today, there are no registry data of HPV-related events and not all morbid events may have a virological origin.

The second limitation relies on the limited dimension of the model. HPV causes a high number of related diseases (cancer of the vulva, vagina, head and neck, etc) [1, 4, 9, 53]. Conversely, the model only considers a limited number of pathological states that consequently underestimate the real impact of HPV-related diseases and the effects of vaccination on Italian women’s health.

The last limitation is related to the methodological assumptions used with reference to transition probabilities. In fact, the model uses an approach for contemporaries on a longitudinal tool [18, 20, 61], assuming that the cohort of individuals recorded in a single year behaves like the generation of vaccinated women. Furthermore, the simplifying assumption of linearity (concerning survivors) and uniform distribution of the events between a specific age and the following one (for example, deaths) should be taken into account. Finally, always considering the estimate method of transition probabilities, the model does not take into account competing risks [20, 23, 62] that may underestimate the real risks of mortality for individuals who avoid disease and death events thanks to vaccination.

## Conclusion

In conclusion, this work is a first attempt to evaluate the actual effect of vaccination on women’s health in Italy by including several HPV-related diseases in addition to cancer morbidity. In our opinion, the model represents a useful tool for measuring the effects of health care intervention on a population, particularlyin the long term. In fact, from this perspective, the impact of HPV vaccination strategies may increase, by affecting the vaccination coverage and the inclusion of males in vaccination programs, but also by developing secondary prevention strategies.

## Additional file

Additional file 1: Unvaccinated and vaccinated cohort. (DOCX 865 kb)

## Abbreviations

AR: Attributable risk
AWS: Anogenital warts
CIN: Cervical Intraepithelial Neoplasia
DALYs: Disability-Adjusted Life Years
HNSCC: Head and neck squamous cell carcinoma
HPV: Human papillomavirus
ISS: National Institute of Health
QALYs: Quality-Adjusted Life Years
RAe: Attributable risk in the exposed
RAp: Attributable risk in the population
RRP: Recurrent respiratory papillomatosis
TTO: Time Trade Off
WHO: World Health Organization
YLD: Years Lost due to Disability
YLL: Years of Life Lost
