# Supplementary material for: Impact of HPV vaccination: health gains in the Italian female population
Source: Popul Health Metr. 2017 Sep 29;15:36. doi: 10.1186/s12963-017-0154-0 (PMC5622511; doi:10.1186/s12963-017-0154-0)
Supplement: Additional file 1: — Unvaccinated and vaccinated cohort. (DOCX 865 kb) [file 12963_2017_154_MOESM1_ESM.docx]

Unvaccinated cohort

Vaccinated cohort:
